# Peripheral Amyloid Precursor Protein Derivative Expression in Fragile X Syndrome

**DOI:** 10.3389/fnint.2019.00049

**Published:** 2019-09-03

**Authors:** Richard D. McLane, Lauren M. Schmitt, Ernest V. Pedapati, Rebecca C. Shaffer, Kelli C. Dominick, Paul S. Horn, Christina Gross, Craig A. Erickson

**Affiliations:** ^1^Division of Child and Adolescent Psychiatry, Cincinnati Children’s Hospital Medical Center, Cincinnati, OH, United States; ^2^Division of Developmental and Behavioral Pediatrics, Cincinnati Children’s Hospital Medical Center, Cincinnati, OH, United States; ^3^Division of Neurology, Cincinnati Children’s Hospital Medical Center, Cincinnati, OH, United States; ^4^Department of Psychiatry and Behavioral Neuroscience, University of Cincinnati College of Medicine, Cincinnati, OH, United States; ^5^Department of Pediatrics, University of Cincinnati College of Medicine, Cincinnati, OH, United States

**Keywords:** amyloid precursor protein, FXS, biomarker, peripheral, enzyme-linked immunosorbent assay

## Abstract

Fragile X syndrome (FXS) is the most common inherited form of intellectual disability and is associated with increased risk for autism spectrum disorder (ASD), anxiety, ADHD, and epilepsy. While our understanding of FXS pathophysiology has improved, a lack of validated blood-based biomarkers of disease continues to impede bench-to-bedside efforts. To meet this demand, there is a growing effort to discover a reliable biomarker to inform treatment discovery and evaluate treatment target engagement. Such a marker, amyloid-beta precursor protein (APP), has shown potential dysregulation in the absence of fragile X mental retardation protein (FMRP) and may therefore be associated with FXS pathophysiology. While APP is best understood in the context of Alzheimer disease, there is a growing body of evidence suggesting the molecule and its derivatives play a broader role in regulating neuronal hyperexcitability, a well-characterized phenotype in FXS. To evaluate the viability of APP as a peripheral biological marker in FXS, we conducted an exploratory ELISA-based evaluation of plasma APP-related species involving 27 persons with FXS (mean age: 22.0 ± 11.5) and 25 age- and sex-matched persons with neurotypical development (mean age: 21.1 ± 10.7). Peripheral levels of both Aβ(1–40) and Aβ(1–42) were increased, while sAPPα was significantly decreased in persons with FXS as compared to control participants. These results suggest that dysregulated APP processing, with potential preferential β-secretase processing, may be a readily accessible marker of FXS pathophysiology.

## Introduction

Fragile X syndrome (FXS) is the most common inherited form of intellectual disability and the most common monogenic cause of autism spectrum disorder (Kosinovsky et al., 2005). FXS is an X-linked disorder affecting 1 in 4,000 males and 1 in 6,000–8,000 females, with all males and some females having significant developmental disability as well as increased risk for autism, anxiety, ADHD, and epilepsy. FXS is caused by a CGG repeat expansion in the promoter region of the fragile X mental retardation 1 gene (*FMR1*), resulting in silencing of the gene and decreased production of fragile X mental retardation protein (FMRP). FMRP is an RNA binding and carrier protein that plays a role in the transport, localization, and translational repression of at least hundreds of target mRNAs (Darnell et al., 2011; Ascano et al., 2012; Westmark, 2018). FMRP-mediated translation is necessary for regulating local protein synthesis and normal cellular processes. When FMRP is absent or expressed at low levels, dendritic spine density and abnormal spine morphology increase, leading to abnormal formation and function of synapses. As a result, neural circuitry is significantly disrupted in individuals with FXS, which is thought to account for the various neurological, behavioral, and behavioral problems associated with this intellectual disability. Although our understanding of FXS pathophysiology has improved, to date, there are still no effective targeted therapies approved in FXS. One of the obstacles preventing the development of disease-modifying treatments for FXS is a lack of useful readily accessible markers of pathophysiology. Biomarkers linked to disease mechanisms may be useful in screening participants, evaluating patient responsiveness to treatment, and identifying subgroups that may best respond to a particular treatment. In recent years, there have been efforts to identify either a single or combination of molecular markers in FXS.

Amyloid precursor protein (APP) is a transmembrane protein with a large extracellular N-terminal domain and a short cytoplasmic tail. Because APP is expressed within microglia, astrocytes, oligodendrocytes, and neurites of the brain and is primarily responsible for cell adhesion and axon pruning (Chasseigneaux and Allinquant, 2012), its regulation is critical to maintaining normal neuronal development and homeostasis (Hartmann et al., 1999; Herms et al., 2004; Guénette et al., 2006; Chasseigneaux and Allinquant, 2012). APP can be metabolized through two distinct processing pathways, the amyloidogenic and non-amyloidogenic processing pathways. In the amyloidogenic processing pathway, APP undergoes cleavage by β-secretase (BACE-1) to produce the neurotoxic amyloid peptides β-amyloid peptides 40 and 42 [Aβ(1–40) and Aβ(1–42)] (Vassar et al., 1999). These peptides are best understood in the context of Alzheimer’s disease where Aβ deposition in brain has been strongly implicated in cerebral plaque formation and brain atrophy (Masters et al., 1985; Hardy and Selkoe, 2002; Persson et al., 2017). However, at lower levels, Aβ monomers are neuroprotective and have been shown to protect mature neurons against excitotoxicity (Whitson et al., 1989). β-cleavage of the soluble N-terminal domain of APP also produces secreted amyloid precursor protein β (sAPPβ) (Vassar et al., 1999). Alternatively, non-amyloidogenic, or α-secretase, processing of APP by two disintegrin and metalloproteases (ADAM-10 and ADAM-17) produces secreted amyloid precursor protein alpha (sAPPα) (Buxbaum et al., 1998; Lammich et al., 1999). Similar to Aβ, sAPPα also has neuroprotective and neurotrophic properties (Mattson et al., 1993; Smith-Swintosky et al., 1994; Luo et al., 2001; Corrigan et al., 2011; Chasseigneaux and Allinquant, 2012). However, less is known regarding altered non-amyloidogenic metabolism.

APP metabolism has been studied in the context of a variety of neurodevelopmental disorders including idiopathic autism, Angelman Syndrome, and FXS (Sokol et al., 2006; Ray et al., 2011; Erickson et al., 2014, 2016; Ray et al., 2016; Westmark et al., 2016b). Previous work has shown that FMRP directly binds and regulates *App* mRNA translation in *FMR1* KO mice (Westmark and Malter, 2007), leading to the potential investigation of APP dysregulation in FXS. In this work, genetic reduction of APP expression in *Fmr1* KO mice has been demonstrated to rescue neuronal hyperexcitability (Westmark et al., 2011b, 2016a), a well-documented neural phenotype in *Fmr1* KO mice, FXS humans, and slice physiology (Gibson et al., 2008; Choi et al., 2015; Ethridge et al., 2016, 2017; Westmark et al., 2016a; Lovelace et al., 2018). Of note, products of both amyloidogenic [Aβ(1–42)] and non-amyloidogenic (sAPPα) APP processing have been shown to enhance mGluR-dependent protein synthesis and contribute to hyperexcitability and altered synaptic plasticity in FXS (Renner et al., 2010; Westmark et al., 2011b, 2016a; Pasciuto et al., 2015). This suggests that targeting the synaptic deficits in FXS via an APP-focused approach may require pharmacotherapeutic manipulation of both amyloidogenic and non-amyloidogenic processing to restore homeostatic levels of APP metabolites (Westmark et al., 2011b, 2016a; Pasciuto et al., 2015).

Peripheral APP metabolite levels also have been reported to be altered in idiopathic ASD and FXS (Sokol et al., 2006; Bailey et al., 2008; Ray et al., 2011, 2016). For example, Ray et al. (2016) reported increased peripheral levels of sAPPα, sAPPβ, sAPP total, Aβ(1–40) and Aβ(1–42) in 18 children with FXS compared to controls. Additionally, increased levels of both sAPPα and total sAPP were found in a small sample of young ASD children with aggressive behavior compared to youth with ASD without aggressive behavior (Bailey et al., 2008). In a follow-up study, children with ASD clinically rated to have severe symptomology based on Childhood Autism Rating Scale (CARS) scores had higher levels of sAPPα than children with ASD who had mild-to-moderate rated symptomology. Additionally, authors reported reduced levels of both Aβ(1–40) and Aβ(1–42) in the more severely affected patient group (Ray et al., 2011). This suggests APP metabolite levels may track with severity of ASD symptoms, and thus may be an important marker of behavioral functioning. Furthermore, in a pilot study of individuals with ASD, our group showed that both sAPPα and sAPP total were reduced in plasma after treatment with acamprosate (Erickson et al., 2014). This suggests the potential utility of APP metabolites as pharmacodynamic markers. Together, initial findings suggest a role for APP metabolites as peripheral biomarkers in neurodevelopmental disorders, though further characterization of peripheral APP metabolites and their association with clinical features are needed in FXS.

In this study, we aimed to add to the existing understanding of peripheral APP expression in FXS by quantifying peripheral APP metabolite and processing enzyme expression in individuals with FXS compared to typically developing controls (TDC). To do so, we conducted a comprehensive evaluation of peripheral APP metabolites including sAPPα, sAPPβ, sAPP total, Aβ(1–40), and Aβ(1–42) and processing enzymes ADAM-10, ADAM-17, and BACE-1 using enzyme-linked immunosorbent assays (ELISA). Finally, we conducted exploratory analyses looking at potential correlations between APP species and APP-associated enzymes and the clinical features of our participants.

## Materials and Methods

### Participant

Plasma samples were collected from 27 individuals with FXS (15 males, 12 females) and 25 age- and sex-matched control subjects (TDC) (14 males, 11 females). Controls had no known prior diagnosis or treatment for developmental or neuropsychiatric disorders. No participant had a history of seizure disorder or current use of anticonvulsant medication, benzodiazepine, or novel potential treatment for FXS (i.e., minocycline, acamprosate, baclofen). All participants completed the Stanford-Binet Intelligence Scale, 5th Edition (SB-5) to assess intellectual functioning. SB-5 standard scores were converted to deviation scores based upon expected age-related performance to estimate intellectual ability in FXS participants for whom reducing floor effects in scores is important (Sansone et al., 2012). All participants or their legal guardians provided informed written consent or verbal assent, when appropriate. The local Institutional Review Board approved the study.

### Blood Sample Collection

Blood samples were collected in 8.5 mL K_2_EDTA tubes (BD Medical, 362799). Plasma samples were prepared within 1-hour post-collection. Plasma was separated from whole blood by centrifuging at 1100 × *g* for 15 min. The isolated plasma was transferred in 2 mL aliquots into several microfuge tubes and flash frozen. The samples were stored at −80°C until analysis.

### Plasma Preparation

Prior to testing, the plasma samples were thawed and filtered through Corning^®^ Costar^®^ Spin-X^®^ centrifuge tube filters (Corning 8163) to remove excess lipids and contaminants. Similar to previous studies (Ray et al., 2011, 2016), we found that immunodepletion of human serum albumin (HSA) improved the detection of sAPPα in plasma (data not shown). HSA was removed from plasma samples using EZAlbumin Depletion Spin Columns (BioVision, Inc., K6573). This immunosubtraction was only performed on samples used for sAPPα.

### ELISA

The concentrations of sAPPα, sAPPβ, total sAPP, Aβ(1–40), Aβ(1–42), ADAM-10, ADAM-17, BACE-1, were quantified through commercially available ELISA kits from IBL America (Catalog# 27734, 27732, 27731, 27718, 27719), LifeSpan Biosciences (LS-F23768), Invitrogen Life Technologies (EHADAM17), and Biomatik (EKU02709). Samples were run according to manufacturer instructions. The assays were run over three consecutive days. On the first day, sAPPα, sAPPβ, total sAPP, Aβ(1–40), and Aβ(1–42) were prepared and allowed to incubate overnight at 4°C. The assays were completed and analyzed the following day. The third day was used to run the remaining moieties: ADAM-10, ADAM-17, and BACE-1. These assays used a biotin-streptavidin detection system that allowed for the tests to be setup and completed all within the same day. Aliquots were stored at 4°C during the 3-day period to prevent protein degradation from repeated freeze-thaw cycles. These storage conditions were tested for each moiety prior to running the experiment. In pilot experiments, no degradation of metabolites was observed up to 5 days in storage at 4°C (data not shown), confirming that these storage conditions adequately maintained sample integrity.

Ideal dilution factors were optimized for each test to allow for consistent and reproducible detection of each analyte. The dilution factors and lower limit of detection (LLOD) for each assay can be found in Supplementary Table S1.

Each sample was run in triplicate at the two dilutions for each analyte. The absorbance for each assay was measured using the Cytation^TM^ 3 plate reader and Gen5^TM^ software from BioTek Instruments, Inc. The standard curve for each assay was modeled with a 5-parameter fit, and the concentrations of the samples were calculated using this model. To limit variability, samples with a coefficient of variation exceeding 10 percent were either rerun to obtain an acceptable value or were excluded from the final analysis [sAPPα (2), ADAM-10 (Darnell et al., 2011), Aβ(1–42) (Kosinovsky et al., 2005), sAPPβ (Darnell et al., 2011), ADAM-17 (4), and BACE-1 (Ascano et al., 2012)].

### Statistical Modeling

An Analysis-of-Covariance (ANCOVA) model was conducted where each amyloid was the response and diagnosis group (FXS vs. TDC) was the independent variable of interest. Covariates included sex, age, and sex^∗^group interaction. Outliers determined by the ROUT method (*Q* = 1%) were excluded from the analysis using GraphPad Prism version 8.01 for Windows, GraphPad Software, La Jolla, CA, United States^1^ (Motulsky and Brown, 2006). Adjusted least-square means (LS means) were derived to compare group effects, or group^∗^sex effects if the interaction term was significant. Lastly, Spearman correlation coefficients, corrected for age, were derived between the amyloid responses and FXS behavior scales for the FXS group. Consistent with prior studies (Ashwood et al., 2010; Ray et al., 2016) and the exploratory nature of this current study, correction multiple testing was not completed. Standard deviations are not available for the generalized linear models conducted here. However, pseudo-effect sizes (d^∗^) may be derived by multiplying the resulting t-statistic (absolute value) for the LS mean differences by the square root of (1/n_1_ + 1/n_2_), where n_1_ and n_2_ are the sample sizes of the two groups being compared. All statistical analyses (except for the outlier detection) were conducted using SAS^®^ version 9.4 (SAS Institute Inc., Cary, NC, United States).

## Results

### Patient Demographics

Results are summarized in Table 1. Subject groups were comprised of 27 FXS (15 males; mean age: 20.5 ± 11.6 years; range: 5.9–40.9, 12 females; mean age: 23.8 ± 11.5 years; range: 8.0–42.9) and 25 age- and sex- matched neurotypical controls (14 males; mean age: 20.4 ± 11.1; range: 5.9–43.5, 11 females; 22.0 ± 10.7; range: 8.1–39.8). FXS participants were significantly more impacted (Deviation IQ = 54.0 ± 29.0; range: 2.3–98.9) than controls (Deviation IQ = 110.5 ± 6.5; range: 90.8–113.7) (*p* < 0.001). Females with FXS were (Deviation IQ = 66.7 ± 24.2; range 23.9–98.9) generally, but not significantly, higher functioning as compared to males with FXS (Deviation IQ = 44.1 ± 20.9; range 2.3–94.1) (*p* = 0.879). All except three males with FXS were full mutation. Two male mosaics were high functioning with deviation IQ scores greater than 90. However, these individuals were not found to impact the results observed.

**TABLE 1:** Characterization of FXS and control subjects.

**Group**	**No.**	**Age**	**Age range**	**IQ**	**IQ range**
**Male**					
FXS	15 (3 mosaic)	20.5 ± 11.6	5.9–40.9	44.1 ± 29.3	2.3–94.1
TDC	14	20.4 ± 11.1	5.9–43.5	101.5 ± 8.2	90.8–113.7
**Female**					
FXS	12	23.8 ± 11.5	8.0–42.9	65.9 ± 22.2	23.9–98.9
TDC	11	22.0 ± 10.7	8.1–39.8	99.6 ± 3.6	95.5–107.4

*Age ranges and IQ scores were measured for both FXS and control subjects.*

### APP Metabolites Are Differentially Expressed in FXS

**FIGURE 1:**
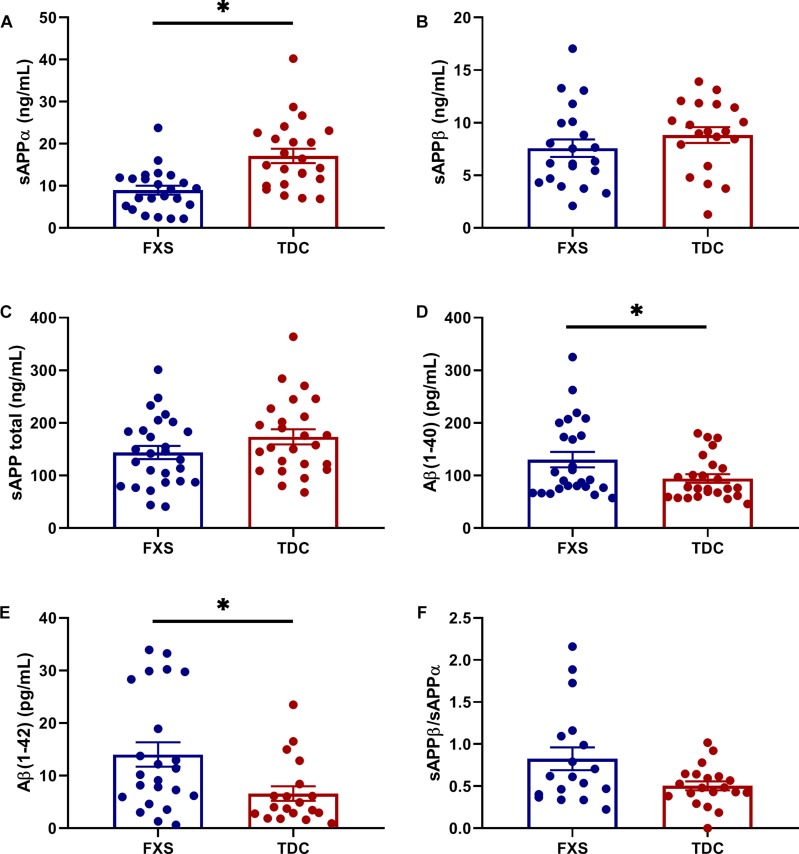
Expression of APP metabolites in plasma from FXS and TDC subjects. Plasma levels of sAPPα, sAPPβ, sAPP total (α and β), Aβ (1–40), and Aβ (1–42) were measured using ELISA in both FXS and TDC participants. Outliers determined by the ROUT method were excluded from analysis [sAPPα (FXS = 3, TDC = 1), sAPPβ (FXS = 2, TDC = 1), Aβ(1–42) (FXS = 1, TDC = 3)]. **(A)** sAPPα was found to be significantly decreased in subjects with FXS as compared to controls (*p* = 0.0003). **(B,C)** Neither sAPPβ nor sAPP total levels were found to be significantly different between groups. **(D,E)** Both Aβ(1–40) and Aβ(1–42) were significantly increased in subjects with FXS as compared to controls (*p* = 0.0169 and 0.0098). **(F)** No significant difference was observed in the ratio of sAPPβ/sAPPα. ^∗^*p* > 0.05.

Results are summarized in Supplementary Table S2. Analytes showing differential expression in the FXS group compared to TDC group are described here. sAPPα levels were significantly reduced in FXS relative to TDC (*p* = 0.0003, *d*^∗^ = 1.13). Aβ(1–40) (*p* = 0.0169, *d*^∗^ = 0.70) and Aβ(1–42) (*p* = 0.0098, *d*^∗^ = 0.85) were significantly increased in FXS participants compared to TDC participants. Neither age nor sex differences contributed to these effects. Significant group differences were not observed in the expression of sAPPβ or sAPP total (*p* > 0.05). Additionally, no group difference in the ratio of sAPPβ/sAPPα were noted when evaluating for any differences in the balance of non-amyloidogenic versus amyloidogenic processing of APP (Figure 1). No correlations between APP metabolites were observed (*p* > 0.05, data not shown).

### APP Processing Enzyme Levels Are Unaltered in FXS

**FIGURE 2:**
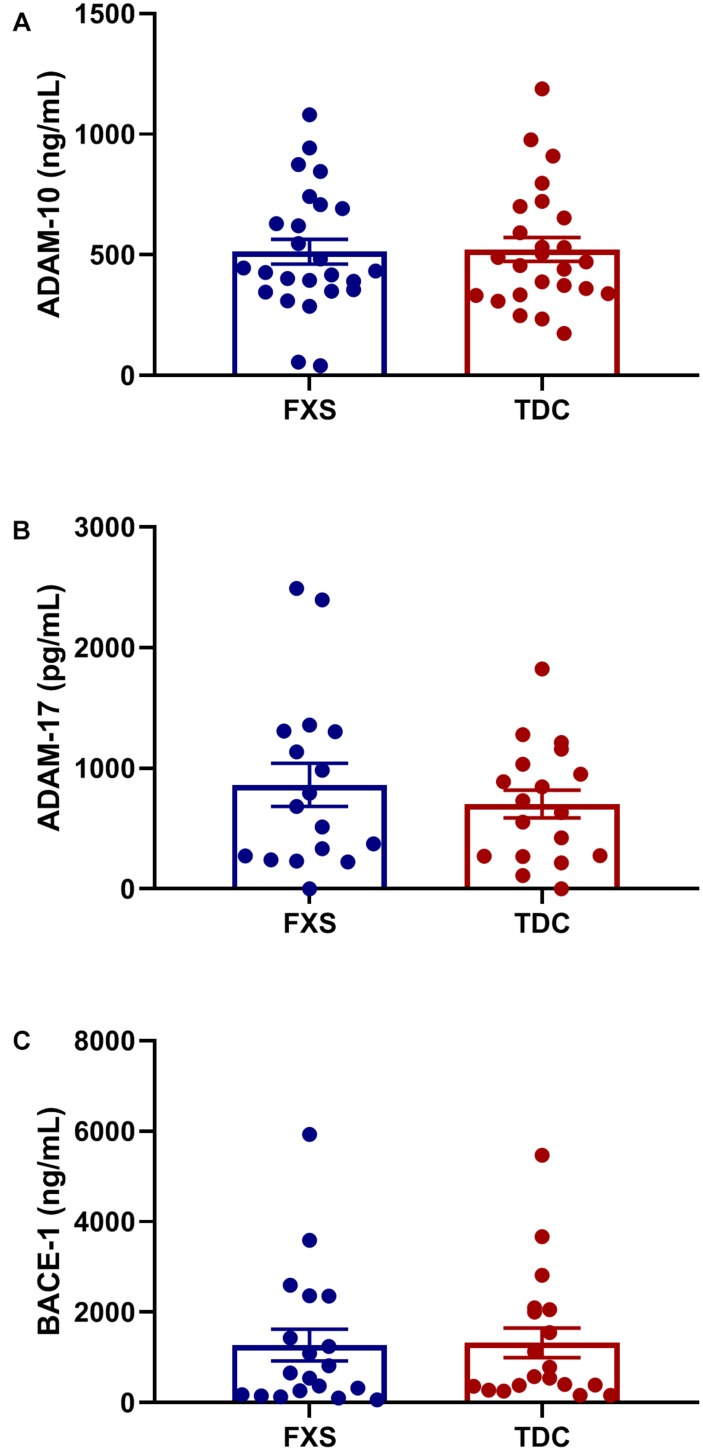
Plasma levels of APP processing enzymes in FXS and control subjects. Plasma levels ADAM-10, ADAM-17, and BACE-1 were measured using ELISA in both FXS and TDC subjects. Outliers determined by the ROUT method were excluded from analysis [BACE-1 (FXS = 3, TDC = 1) and ADAM-17 (FXS = 3, TDC = 3)] **(A–C)** No significant differences were observed in the expression of ADAM-10, ADAM-17, and BACE-1 (*p* > 0.05).

Plasma levels of enzymes contributing to the amyloidogenic (BACE-1) and non-amyloidogenic (ADAM-10 and ADAM-17) were measured to see if differences in APP metabolites could be attributed to abnormal enzyme concentrations. However, no significant differences in total enzyme levels were found between groups (Figure 2). Additionally, no correlations were found between enzyme and metabolite concentrations using generalized mixed linear modeling with lognormal regression (Supplementary Table S3).

### Expression of Metabolites and Enzymes Changes With Age

**FIGURE 3:**
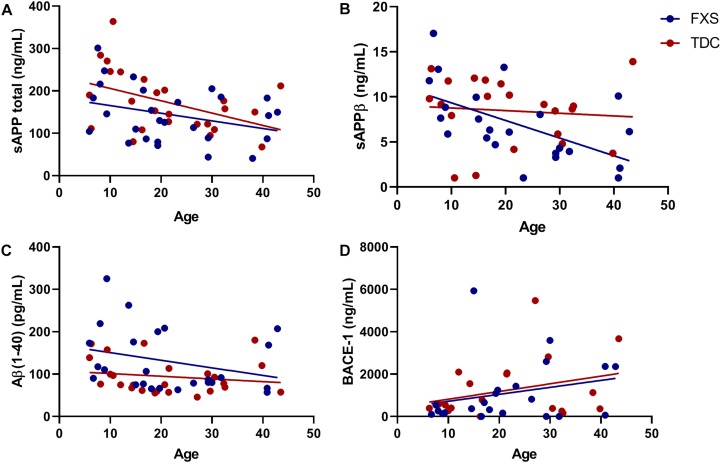
BACE-1 activity may decrease with age. Levels of sAPP total, sAPPβ, Aβ(1−40), and BACE-1 captured by ELISA were analyzed with respect to subject age. **(A)** The expression of sAPP total decreases significantly in both FXS and TDC groups with respect to age (*p* = 0.0112). **(B)** The expression of sAPPβ decreases significantly in both FXS and TDC groups with respect to age (*p* = 0.0074). **(C)** Aβ(1–40) appears to be elevated in younger FXS patients and seems to decrease with age (*p* = 0.0644). **(D)** BACE-1 expression tended to increase with age although this trend did not reach statistical significance (*p* = 0.0548).

The effect of age was analyzed with respect to metabolite and enzyme expression (Figure 3). Both sAPPβ and sAPP total levels were found to significantly decrease with age in both groups (*p* = 0.0074, 0.0112). Similarly, Aβ(1–40) levels were inversely proportional to age (*p* = 0.0644) for both FXS and TDC groups. While both major metabolites of β-cleavage were found to decrease with age, BACE-1 levels appeared to increase with age (*p* = 0.0548) for each group. Neither sex nor mosaicism were found to affect the expression of any of the APP metabolites or enzymes measured.

## Discussion

We report a distinct molecular phenotype in our FXS participants as compared to matched controls with a significant decrease in peripheral levels of sAPPα and significant increases in peripheral levels of both Aβ(1–40) and Aβ(1–42). These results suggest potential preferential amyloidogenic, or β-secretase, processing of APP in individuals with FXS, as found in Alzheimer disease.

Similar to our findings, increased plasma concentrations of Aβ monomers have been previously reported in FXS (Westmark et al., 2011b; Ray et al., 2016). Although excess Aβ(1–40) and Aβ(1–42) are best understood in the context of Alzheimer disease, there are multiple ways that it can contribute to key phenotypes in FXS. In the brain, Aβ can significantly alter the excitability of the system both directly and indirectly. In APP overexpressing hippocampal slice neurons, Aβ has been shown to direct synaptic remodeling and depress excitatory synaptic signaling. Aβ levels also increase or decrease with respective excitation or depression of the neuronal activity and have been suggested to regulate hyperexcitability (Kamenetz et al., 2003). In the context of FXS, increased Aβ monomers may be indicative of a similar compensatory mechanism mediating neuronal hyperexcitability (Gibson et al., 2008; Choi et al., 2015; Ethridge et al., 2016, 2017; Westmark et al., 2016a; Lovelace et al., 2018). In contrast, excessive Aβ can form oligomers that, in conjunction with an extracellular scaffolding protein, can redistribute and reduce lateral mobility of mGluR_5_ receptors, ultimately resulting in increased intracellular Ca^2+^ and neuronal excitation (Renner et al., 2010). Therefore, we speculate that excess Aβ could enhance neuronal excitability and lead to a positive feedback loop that increases hyperexcitability. Together, these findings indicate increased peripheral levels of both Aβ(1–40) and Aβ(1–42) are reflective of hyperexcitability and increased expression of APP and mGluR_5_ in FXS. Thus, Aβ(1–40) and Aβ(1–42) each may be promising biomarkers of neural hyperexcitability in FXS.

Notably, a potential subgroup of FXS participants seem potentially represent a cluster of the highest levels of Aβ(1–42). Future studies including environmental and behavioral analyses may help to determine the cause of increased Aβ(1–42) in these individuals. For example, high fat diets have been show to promote the formation of the BACE-1/Adaptor protein-2/clathrin complex in mice, increasing the amount of intracellular BACE-1 and subsequent cleavage of APP (Maesako et al., 2015). Additionally, different behaviors, such as aggression, also correlate with levels of sAPPα in patients with ASD (Sokol et al., 2006). It could be that environmental or behavioral differences could similarly contribute to differences in peripheral metabolite expression within and between groups.

We also observed a significant reduction in peripheral sAPPα. Since both the α- and β-secretase(s) compete for APP as a substrate, the levels of their respective products also should vary inversely. With increased levels of peripheral Aβ, it is not surprising that there is a significant reduction in sAPPα. Our findings contradict previous reports by Ray et al. (2016) in which sAPPα was found to be increased in the plasma of patients with FXS. While we tested a number of plasma samples from patients with FXS from childhood to adulthood, Ray et al. (2016) only analyzed samples from children. Previous studies have shown that sAPPα is increased in juvenile *FMR1* KO brain at p21, and sAPP total is dysregulated at p21 and p30, but both return to homeostatic levels after these time points (Pasciuto et al., 2015). Restricting participant ages to children within this neurodevelopmental window may better capture potential increases in peripheral sAPPα and sAPP total.

While peripheral levels of APP metabolites were altered, we did not find any differences in the levels of their respective processing enzymes: ADAM-10, ADAM-17, and BACE-1 in FXS compared to TDC. Additionally, no correlations were found between any of the enzyme concentrations and the concentrations of their respective metabolites, importantly suggesting that total peripheral enzyme levels may not be indicative of peripheral metabolite regulation. Indeed, since ADAM-10, ADAM-17, and BACE-1 all act on numerous targets in multiple tissues, their peripheral expression may fluctuate less in response to increased APP (Barão et al., 2016; Moss and Minond, 2017; Wetzel et al., 2017). Clearance of APP metabolites from the brain and other tissues also could strongly influence peripheral metabolite levels, making the direct relationship between concentrations of enzymes and metabolites less accurate. With multiple tissue subtypes contributing to peripheral metabolite concentrations, the lack of correlation between peripheral metabolite and enzyme expression is expected. Additionally, peripheral concentrations may also not be indicative of enzymatic activity. For example, increased peripheral BACE-1 activity could result in higher turnover of sAPPβ to both Aβ peptides. This could potentially account for the differences in metabolite expression, while no differences were observed in enzyme concentration. These enzymes also could be differentially regulated during critical developmental periods not captured within our wider age range of participants. For example, in *FMR1* KO ADAM-10 expression is dysregulated in cortical neurons during a critical neurodevelopmental window in juvenile mice (Pasciuto et al., 2015). Thus, future studies are needed examine processing enzymes in a more restricted range of individuals with FXS.

Interestingly, we noted several molecular changes with age in both persons with FXS and control participants, including sAPP total, sAPPβ, Aβ(1–40), and BACE-1. Given associations were observed across both patient and control participants, this suggests that potentially developmental changes of APP metabolites and enzyme concentrations is intact in FXS. Since sAPP total is a total measure of sAPPα and sAPPβ, its significant decrease with respect to age can largely be attributed to the decrease in sAPPβ levels. Counterintuitively, while peripheral expression of both Aβ(1–40) and sAPPβ decrease with age, BACE-1 levels increase with age in both our FXS and control groups. The inverse relationship between amyloidogenic metabolites and BACE-1 reinforces that there is no clear relationship between peripheral metabolite and enzyme levels in FXS.

The results of our work should be understood within the context of the limitations of our experimental design. The greatest limitation was the overall sample size. With the significant variability of multiple metabolites with respect to age, it is possible that more subtle differences in metabolite and enzyme expression may have been captured within a narrower age range and/or a larger sample size. Correlations with clinical features such as IQ may have also been limited by sample size. Given the potential utility of APP metabolites as peripheral biomarkers in FXS, future studies including with larger participant pools need to be completed to evaluate for correlations with clinical data. Additional measures of clinical severity were not available to evaluate correlations with APP metabolites. Future work with an expanded number of subjects and deeper phenotyping will aide these efforts.

Amyloid-beta precursor protein metabolite concentrations also have a diurnal expression pattern in both cerebrospinal fluid and blood (Dobrowolska et al., 2014). Since not all blood was collected at the same time of day, relative levels of APP within participants may vary which could either prevent us from observing a small effect or lead us to observing an exaggerated effect. Additionally, blood was collected in tubes using K_2_EDTA as a preservative, which has been shown to significantly reduce levels of Aβ(1–42) in plasma (Westmark et al., 2011a). Because K_2_EDTA was used to collect all samples, the effect size of differences in Aβ(1–42) levels between groups may have been underestimated in this study.

We also report no differences in metabolite or enzyme expression between males, full mutation and mosaic, and females with FXS (Supplementary Table S2), which is somewhat unexpected. Many of the effects are subtle and may require a more sensitive platform to detect and/or larger subject cohorts to discern potentially more subtle differences. Additionally, our FXS female sample did not differ on IQ from their male counterparts, suggesting FXS males and females were similarly affected in the current study. Thus, it is possible with a more representative FXS female sample, sex differences in primary measures may emerge. Last, we are using peripheral APP metabolite and enzyme levels as a proxy to evaluate their relative expression in brain. To date, there are a very limited amount of known proteins that are expressed in parallel between brain and blood (Tajima et al., 2013). In addition to the brain, APP is also expressed in the thymus, heart, muscle, lung, kidney, adipose tissue, liver, spleen, skin, and intestine (Beer et al., 1995). Similarly, the processing enzymes are also expressed in a variety of different tissue types. Thus, blood levels of APP metabolites are most likely influenced by their expression in many organs of the body. This makes comparing peripheral APP levels to levels observed in the brain much more difficult and introduces a level of uncertainty to the measures.

## Conclusion

In conclusion, we determined a distinct molecular pattern of APP metabolite expression with increased Aβ(1–40) and Aβ(1–42) and decreased sAPPα. While we suggest that there is increased β-secretase activity in FXS, more work needs to be completed to determine the exact mechanisms leading to increased peripheral Aβ. Still our findings provide new evidence of the promising potential of APP metabolite expression as a blood-based biomarker in FXS. Ultimately, our work highlights the need for more thorough characterization of APP expression patterns with both behavioral and electrophysiological patterns in FXS, which may provide additional insight into the mechanistic roles of APP metabolites.

## Data Availability

The datasets generated for this study are available on request to the corresponding author.

## Ethics Statement

All subjects gave written informed consent in accordance with the Declaration of Helsinki. The protocol was approved by the Cincinnati Children’s Hospital Institutional Review Board.

## Author Contributions

RM aided in the study design, responsible for collecting, analyzing, and interpreting the molecular data, and writing of the manuscript. LS contributed significantly to the manuscript preparation and deviation IQ analyses. EP and KD contributed significantly to the study setup and design. RS responsible for collecting and interpreting the clinical measures. PH performed all the statistical modeling and analyses. CG aided in the study design and contributed significantly to the molecular analysis and interpretation. CE significantly contributed to the study setup and design, as well as manuscript preparation. All authors contributed substantially to the study, and read and approved the final version of the manuscript.

## Conflict of Interest Statement

RS receives funding from the Fulcrum Therapeutics. CE has received current or past funding from the Confluence Pharmaceuticals, Novartis, F. Hoffmann-La Roche Ltd., Seaside Therapeutics, Riovant Sciences, Inc., Fulcrum Therapeutics, Neuren Pharmaceuticals Ltd., Alcobra Pharmaceuticals, Neurotrope, Zynerba Pharmaceuticals, Inc., Lenire Bioscience, and Ovid Therapeutics Inc., to consult on trial design or development strategies and/or conduct clinical trials in FXS or other neurodevelopmental disorders and he is additionally the inventor or co-inventor on several patents held by the Cincinnati Children’s Hospital Medical Center or Indiana University School of Medicine describing methods of treatment in FXS or other neurodevelopmental disorders. EP has received research support by the National Institutes of Health (NIMH), American Academy of Child and Adolescent Psychiatry, and Cincinnati Children’s Hospital Research Foundation and he is a clinical trial site investigator for the Marcus Autism Center (clinical trial, Autism), he receives compensation for consulting for Proctor & Gamble and Eccrine Systems, LLC and also receives book royalties from the Springer. There are no conflicts of interest with the current manuscript. KD has received research support from the National Institute of Neurological Disorders and Stroke (NINDS), American Academy of Child and Adolescent Psychiatry, and Cincinnati Children’s Hospital Medical Center and she is a clinical trial site investigator for F. Hoffman-La Roche Ltd., and Ovid Therapeutics. There are no conflicts of interest for the current manuscript. CG currently receives funding from NICHD, NINDS, and the Brain & Behavior Research Foundation and has received funding from NIMH, NFXF, FRAXA, and the Epilepsy Foundation in the past. There are no conflicts of interest for the current manuscript. The remaining authors declare that the research was conducted in the absence of any commercial or financial relationships that could be construed as a potential conflict of interest.

## Footnotes

1 www.graphpad.com

## Supplementary Material

TABLE S1 Dilution factors and lower limits of detection for each ELISA. Prior to analysis, all analyte concentrations were optimized per ELISA plate. All samples were run with the dilutions listed in the table.

TABLE S2 Analytes only significantly varied with respect to group. Levels of all analytes were captured by ELISA and analyzed with respect to group. Only sAPPα, Aβ(1–40), and Aβ(1–42) were significantly different between groups. No significant Sex^∗^Group interactions were found for any of the analytes tested (*p* > 0.05).

TABLE S3 Metabolite concentrations did not correlate significantly with their respective processing enzymes. The relationship between levels of APP metabolites and enzymes were examined with respect to group using generalized linear models with lognormal regression. No correlates were found to be significant (*p* > 0.05).
