# Supplementary material for: Peripheral Amyloid Precursor Protein Derivative Expression in Fragile X Syndrome
Source: Front Integr Neurosci. 2019 Sep 3;13:49. doi: 10.3389/fnint.2019.00049 (PMC6733993; doi:10.3389/fnint.2019.00049)
Supplement: TABLE S1 — Dilution factors and lower limits of detection for each ELISA. Prior to analysis, all analyte concentrations were optimized per ELISA plate. All samples were run with the dilutions listed in the table. [file Data_Sheet_1.docx]

**Supplemental Table 1**

| **Analyte** | **Dilution Factors** | **Lower Limit of Detection** |
| --- | --- | --- |
| sAPPα | 2, 4 | 0.78 ng/mL |
| sAPPβ | 2, 4 | 0.78 ng/mL |
| sAPP total | 10, 20 | 0.39 ng/mL |
| Aβ(1-40) | 4, 8 | 1.56 pg/mL |
| Aβ(1-42) | 2, 4 | 1.56 pg/mL |
| ADAM-10 | 600, 1200 | 62.5 pg/mL |
| ADAM-17 | 2, 4 | 78.15 pg/mL |
| BACE-1 | 200, 800 | 0.25 ng/mL |

**Supplemental Table 2**

| **Analyte** | **FXS Average** | **TDC Average** | **p-value** | **Effect Size (d*)** | **Sex*Group p-value** |
| --- | --- | --- | --- | --- | --- |
| sAPPα (ng/mL) | 8.97 ± 5.09 | 17.10 ± 8.132 | *0.0003 | 1.13 | 0.7979 |
| sAPPβ (ng/mL) | 7.58 ± 3.82 | 8.82 ± 3.36 | 0.1745 | 0.42 | 0.3411 |
| sAPP total (ng/mL) | 143.70 ± 64.98 | 173.50 ± 71.28 | 0.1148 | 0.45 | 0.5510 |
| Aβ(1-40) (pg/mL) | 130.20 ± 72.85 | 94.27 ± 40.69 | *0.0169 | 0.70 | 0.2790 |
| Aβ(1-42) (pg/mL) | 14.02 ± 11.10 | 6.602 ± 6.090 | *0.0098 | 0.85 | 0.6190 |
| ADAM-10 (ng/mL) | 512.676 ± 255.938 | 522.244 ± 248.440 | 0.5451 | 0.17 | 0.0701 |
| ADAM-17 (pg/mL) | 861.6 ± 737.5 | 704.5 ± 486.5 | 0.9658 | 0.01 | 0.6189 |
| BACE-1 (ng/mL) | 1274 ± 1523 | 1321 ± 1424 | 0.8716 | 0.04 | 0.5637 |

**Supplemental Table 3**

|  |  | **FXS** | | **TDC** | |
| --- | --- | --- | --- | --- | --- |
| **Enzyme** | **Metabolite** | **No.** | **p-value** | **No.** | **p-value** |
| ADAM-10 |  |  |  |  |  |
|  | sAPPα | 24 | 0.185 | 24 | 0.912 |
| ADAM-17 |  |  |  |  |  |
|  | sAPPα | 15 | 0.612 | 19 | 0.176 |
| BACE-1 |  |  |  |  |  |
|  | sAPPβ | 17 | 0.794 | 20 | 0.923 |
|  | Aβ(1-40) | 20 | 0.794 | 22 | 0.275 |
|  | Aβ(1-42) | 18 | 0.999 | 17 | 0.923 |
